# Evaluation of Immunoreactivity and Protection Efficacy of Seneca Valley Virus Inactivated Vaccine in Finishing Pigs Based on Screening of Inactivated Agents and Adjuvants

**DOI:** 10.3390/vaccines10040631

**Published:** 2022-04-18

**Authors:** Wenqiang Liu, Xiangmin Li, Huawei Zhang, Genxi Hao, Xianfei Shang, Huilan Wang, Huanchun Chen, Ping Qian

**Affiliations:** 1 State Key Laboratory of Agricultural Microbiology, Huazhong Agricultural University, Wuhan 430070, China; liuwenqiang1993@webmail.hzau.edu.cn (W.L.); lixiangmin@mail.hzau.edu.cn (X.L.); zhanghuawei@webmail.hzau.edu.cn (H.Z.); haogenxi@webmail.hzau.edu.cn (G.H.); shangxf@webmail.hzau.edu.cn (X.S.); wanghuilan@webmail.hzau.edu.cn (H.W.); chenhch@mail.hzau.edu.cn (H.C.); 2 College of Veterinary Medicine, Huazhong Agricultural University, Wuhan 430070, China; 3 Key Laboratory of Development of Veterinary Diagnostic Products, Ministry of Agriculture, Wuhan 430070, China; 4 Key Laboratory of Preventive Veterinary Medicine in Hubei Province, The Cooperative Innovation Center for Sustainable Pig Production, Wuhan 430070, China

**Keywords:** Seneca Valley virus (SVV), inactivated vaccine, β-propiolactone (BPL), MONTANIDE^TM^ IMG 1313 VG N (1313), protection efficacy

## Abstract

Seneca Valley virus (SVV), also known as Senecavirus A (SVA), is a non-enveloped and single-strand positive-sense RNA virus, which belongs to the genus of *Senecavirus* within the family *Picornaviridae*. Porcine idiopathic vesicular disease (PIVD) caused by SVV has frequently been prevalent in America and Southeast Asia (especially in China) since the end of 2014, and has caused continuing issues. In this study, an SVV strain isolated in China, named SVV LNSY01-2017 (MH064435), was used as the stock virus for the preparation of an SVV-inactivated vaccine. The SVV culture was directly inactivated using binary ethyleneimine (BEI) and β-propiolactone (BPL). BPL showed a better effect as an SVV inactivator, according to the results of pH variation, inactivation kinetics, and the detection of VP1 content during inactivation. Then, SVV inactivated by BPL was subsequently emulsified using different adjuvants, including MONTANIDE^TM^ ISA 201 VG (ISA 201) and MONTANIDE^TM^ IMG 1313 VG N (IMS 1313). The immunoreactivity and protection efficacy of the inactivated vaccines were then evaluated in finishing pigs. SVV-BPL-1313 showed a better humoral response post-immunization and further challenge tests post-immunization showed that both the SVV-BPL-201 and SVV-BPL-1313 combinations could resist challenge from a virulent SVV strain. The SVV LNSY01-2017-inactivated vaccine candidate developed here represents a promising alternative to prevent and control SVV infection in swine.

## 1. Introduction

SVV is a non-enveloped, single-stranded positive-sense RNA virus and the only species of the genus *Senecavirus* within the family of *Picornaviridae* [1]. Similar to other *Picornaviridae* viruses, the polypeptide precursor protein of SVV is cleaved into four structure proteins and eight non-structure proteins [1]. A previous study [2] suggested that VP2 and VP3, the structure proteins of SVV, are likely to be the main binding sites of antigen epitopes during the SVV infection, and neutralizing antibody (NA) responses were strongly correlated with VP2- and VP3-specific IgM responses. Furthermore, it has been established that there are a total of six liner B cells epitopes on VP1 (1) and VP2 (5) proteins using monoclonal antibodies [3].

In 2007, a pig farm in Canada reported the appearance of PIVD caused by SVV [4]. At the end of 2014, Brazil reported an infection by SVV; it seems that the spread of SVV has been accelerated [5]. During 2015, at least seven regions experienced SVV infections among pigs of various ages in Brazil, including piglets, which was the first case of clinical manifestation in piglets that has been reported. Considering the cases above, there seems to have been an evolution of SVV into a virulent phenotype [6,7,8]. In China, 2016 is considered to have been a turning point for SVV epidemiology. Two subclades of SVV were idetified in China; the strains of SVV isolated before 2016 have a high nucleotide homology with the strains from Canada and Brazil, while the strains of SVV in China after 2016 were more closely related to those found in the United States [5,9,10,11,12,13]. Up to now, outbreaks of PIVD caused by SVV have been reported in most provinces of China. In recent years, some laboratory test methods have been developed that can detect SVV infection, such as iELISA, cELISA, virus neutralization test assay, and reverse transcription polymerase chain reaction (RT-PCR) [14,15,16,17,18]. However, there are no commercial vaccines that can inoculate against SVV infection. In 2018, Yang developed an SVV-inactivated vaccine candidate, which used the virus produced by a BHK-21 cell culture, derived for purity, so that can be used to prepared an inactivated vaccine. Pigs immunized with the full dose of the inactivated vaccine could completely defend against challenge by SVV [19]. Yang selected BEI as the inactivator, but details regarding inactivation were not fully shown. In Yang’s study, he used a purified SVV virus as an immunogen. Purifying a virus is beneficial to enrich antigens. However, the purification method employed by Yang may have had some disadvantages, including being time-consuming and scale-restricted, with viral infectivity also being lost. Our study aimed at developing a more convenient preparation method, which preserves specific antigens as much as possible, and provide more-specific inactivation details. The inactivators and adjuvants, which are more suitable for preparing SVV-inactivated vaccine, were further screened.

### 1.1. Cells, Virus and Animals

Baby hamster kidney cells (BHK-21 cells; ATCC, CCL-10) were grown at 37 °C in Dulbecco’s modified essential medium (HyClone, SH30022.01) containing 10% FBS (Gibco, 16000044) in a humidified 5% CO_2_ incubator. A total of 100 U/mL of penicillin and 100 µg/mL of streptomycin (GENVIEW, GA3502) were also added to the culture media. The SVV LNSY01-2017 (MH064435) strain was isolated by our laboratory from a vesicular lesion swab collected from a finishing pig. The virus was inoculated on the monolayer of BHK-21 cells, and cytopathic effects (CPE) were observed daily under a microscope. Culture supernatants were harvested and re-inoculated on fresh BHK-21 cells until a typical SVV CPE appeared. The viral titer was determined from BHK-21 cells using the median tissue culture infective dose (TCID_50_) and the pathogenicity of SVV LNSY01-2017 was also confirmed in the finishing pigs. 

Fifteen large white growing finishing pigs, weighing 25–35 kg, were purchased from the experimental farm of Huazhong Agricultural University and were randomly divided into three groups: the SVV-BPL-201 and SVV-BPL-1313 groups, and the DMEM group which served as the negative control. All large white growing finishing pigs (castrated hogs) were confirmed to be seronegative for SVA via neutralization assay and RT-PCR assays.

### 1.2. Western Blotting

BHK-21 cells were infected with the SVV LNSY01-2017 strain. At 18 h post-infection, cells were collected and then 80 µL of NP40 lysate (Shanghai Chuntest Biotechnology Co. Ltd., Shanghai, China) was added and incubated at a low temperature for 45 min. Samples were centrifuged at 12,000 rpm for 10 min. The unpurified protein from the cell lysate supernatant was collected. The protein concentrations of cell lysate supernatants were measured using a bicinchoninic acid protein assay kit (Thermo Scientific, Rockford, IL, USA). Equal amounts of proteins were analyzed using Western blotting. The equal-volume sampling collections were predetermined as inactivated and collections from different days post inactivation were added to a 2× concentration of loading buffer and then boiled for 10 min. The protein samples were separated using 12% sodium dodecyl sulfate polyacrylamide gel electrophoresis (SDS-PAGE) and then transferred onto polyvinylidene difluoride membranes. The membranes were blocked using blocking buffer (5% skimmed milk in Tris-buffered saline and Tween 20 (TBS-T)) at 4 °C, overnight, or at RT for 2 h). The membranes were subsequently incubated with diluted primary antibodies at RT for 1.5 h, or at 4 °C overnight, respectively. HRP-conjugated anti-rabbit or anti mouse IgGs were used as secondary antibodies. An enhanced chemiluminescence substrate was used for detection (Thermo Scientific, Carlsbad, CA, USA). All immunoblot images were obtained using a Bio-Rad ChemiDoc XRS+ instrument and image software. (Bio-Rad, Hercules, CA, USA) Densitometric analysis of each band was performed using ImageJ software to analyze the change in VP1 contents of different inactivated collections. Expression of alpha tubulin was assessed using anti-alpha tubulin monoclonal antibodies and was used as an internal reference.

### 1.3. Immunofluorescence Assay 

BHK-21 cells were infected with the SVV LNSY01-2017 at a MOI of 1 in 24-well plates. After 24 h, the cells were fixed with methanol/acetone (1:1) for 15 min at −20 °C. Subsequently, cells were incubated with 2% bovine serum albumin (BSA). The cells were incubated with anti-SVV VP1 polyclonal antibody (1:100 dilution; homemade) for 1.5 h at 37 °C. After washing three times with PBS, cells were incubated with Goat anti-Rabbit IgG (H+L) Highly Cross-Adsorbed Secondary Antibody, Alexa Fluor 488 (1:1000 dilution; Invitrogen, Eugene, OR, USA) for 1 h at 37 °C. The cells were observed under an inverted fluorescence microscope (Nikon, Tokyo, Japan).

### 1.4. Virus Inactivation

BEI and BPL were used for virus inactivation and were diluted (*v/v*) with bacteria-free PBS to achieve final concentrations of 0.625 mM, 1.00 mM, 1.25 mM, and 2.50 mM for BEI, and 0.1%, 0.3%, 0.5%, and 0.7% for BPL. Different concentrations of BEI were incubated with SVV at 30 °C and were collected after 1, 2, 4, 8, 10, 12, 16, 20, 36, and 48 h. Samples collected at 1, 2, 4, 8, 10, 12, 16, and 24 h were selected to determine the inactivation curve. Then, the reactions were stopped by adding sterilized 0.2% sodium thiosulfate at 37 °C for 0.5, 1, and 2 h. Different concentrations of BPL were incubated with SVV at 4 °C and collected after 1, 2, 4, 8, 10, and 16 h. After inactivation, the reactions were stopped in water bath at 37 °C for 0.5, 1, and 2 h.

### 1.5. Preparation of Inactivated Vaccines

The inactivated virus preparation deemed to be optimal (as determined using the method above) was added to either equal volumes of MONTANIDE^TM^ ISA 201 VG or MONTANIDE^TM^ IMS 1313 VG (SEPPIC, Paris, France) following the recommendations from SEPPIC. The prepared inactivated vaccine was stored at 4 °C and shaken well before use.

### 1.6. Vaccine Immunization of Pig

Fifteen 6-week-old pigs that were negative for both PCR and the neutralizing antibody of SVV were selected. The pigs were randomly divided into 3 groups (n = 5): SVV-BPL-201 and SVV-BPL-1313 groups, and the DMEM group as a negative control. Pigs from the individual groups were injected intramuscularly (into the back) with 2 mL (1 dose) of the corresponding vaccine. Booster immunization was performed using the same dosage, 28 days after the first immunization. 

### 1.7. Samples Collection

Whole blood was collected at 0, 14, 21, 28, 35, 42, and 49 days to detect neutralizing antibody titers, from the first immunization to the end of the immunization period (49 days). During the protection challenge period, whole blood was collected every 2 days. Blood was used to extract RNA for the analysis of viremia using qRT-PCR. Eight days post SVV challenge, pigs showing typical symptoms of SVV were randomly selected from the control group, and pigs selected at random from the vaccine groups were euthanized. Spleen, mesenteric lymph node, inguinal lymph node, snout, and coronary band were selected for histopathological observation.

### 1.8. Neutralization Assay 

A neutralization assay was conducted as previously described [20]. Briefly, the inactivated pig serum (56 °C, 30 min) was serial-diluted, and then mixed with 50 µL of 200 TCID_50_ SVV LNSY01-2017 virus at 37 °C for 90 min. Serum–virus mixtures were added to confluent BHK-21 cells cultured in 96-well plates and then incubated at 37 °C for 4 days. Neutralizing antibody titers against the SVV LNSY01-2017 strain were calculated and expressed as the log2 of the reciprocal of the highest serum dilution that inhibits 100% of SVV infection/replication in the culture wells. 

### 1.9. Quantitative Real-Time PCR 

Quantitative real time polymerase chain reaction (qRT-PCR) was performed, as previously described [16,20]. The 3D SVV primers (SVV 3D-F: 5′-AGAATTTGGAAGCCATGCTCT-3′; SVV 3D-R:5′-GAGCCAACATAGATACAGATTGC-3′) were synthesized and the TaqMan probe was 5′-FAM-TTCAAACCAGGAACACTACTCGAG-TAMRA-3′. RNA was extracted from the samples using Trizol reagent (Invitrogen, Carlsbad, CA, USA). One microgram of total RNA was reverse transcribed in a 20 µL volume of HiScript III RT SuperMix +gDNA wiper (Vazyme, Nanjing, China) for qPCR, according to the manufacturer’s protocols. qRT-PCR amplifications were performed using a CFX96 Touch RT-PCR Detection System (Bio-Rad, Hercules, USA). Reaction mixtures contained cDNA (20–100 ng), SuperReal PreMix (probe) (Bio-Rad, Hercules, USA), TaqMan probe (Sangon Biotech, Shanghai, China), sense and reverse primers (20 µmol/L) (Sangon Biotech, Shanghai, China), and RNase-free water at a total volume of 20 µL. The PCR cycling conditions were as follows: initial denaturation at 50 °C for 2 min and at 95 °C for 10 min; followed by 40 cycles of 15 s at 95 °C, 30 s at 60 °C, and 30 s at 72 °C. Viral genome copy numbers were determined using a standard curve, and results were expressed as log10 RNA copies/mL. The data represent results from one representative triplicate experiment. 

### 1.10. Histopathological Examination 

At 8 dpc, three pigs (one selected from each group) were euthanized. During necropsy, organs were collected and subjected to pathological examination. Collected samples were fixed in 10% PBS buffered formalin for 24–36 h, dehydrated with different ethanol concentrations, and the fixed in paraffin and sectioned. HE staining was performed on thin sections (3–6 µm thickness). 

### 1.11. Statistical Analysis 

Statistical analyses were performed using GraphPad Prism software 8.0 (GraphPad Software Inc., La Jolla, CA, USA). Data were expressed as the mean ± standard deviation (SD). Significance was determined using one-way ANOVA (* *p* < 0.05; ** *p* < 0.01; *** *p* < 0.001, and **** *p* < 0.000001).

## 2. Results

### 2.1. Characteristics of SVV LNSY01-2017 on BHK-21 Cells

**Figure 1:**
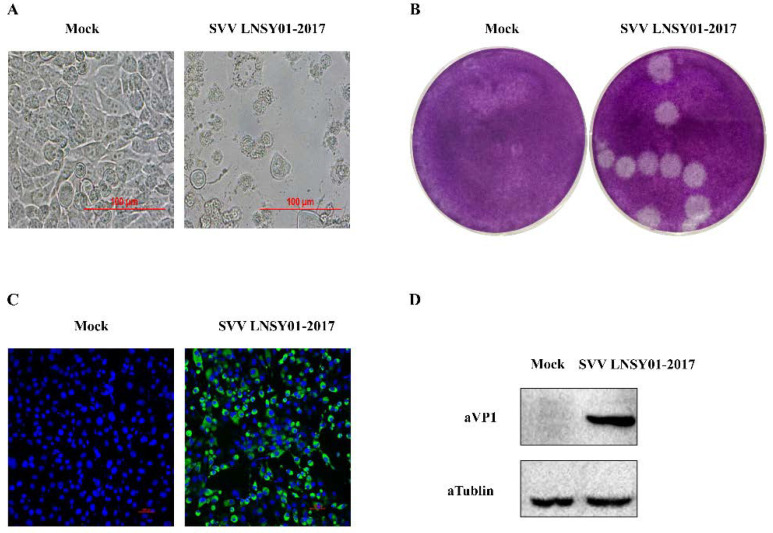
Characteristics of SVV LNSYO1-2017. (**A**) The cytopathic effect of BHK-21 cells infected with SVV LNSYO1-2017 strain at 24 h post-infection. (**B**) Plaque morphology in BHK-21 cells infected with third-passage of SVV LNSYO1-2017 strain at 48 h post-infection. (**C**) Immunofluorescence assay (IFA) of BHK-21 cells infected with SVV LNSYO1-2017 strain at 18 h post-infection. Cells were stained with primary antibody using homemade rabbit anti-SVV VP1 polyclonal antibody. (**D**) Western blotting analyses of BHK-21 cells infected with SVV LNSYO1-2017 strain at 18 h post-infection. Cells were stained with primary antibody using a homemade rabbit polyclonal anti-SVV VP1 antibody and mouse anti-tubulin antibody.

Based on the SVV LNSY01-2017 strain, which was previously isolated and identified as highly pathogenic, we selected strains as the inactivated vaccine stock. We evaluated the characteristics of SVV LNSY01-2017 infection of BHK-21 cells. The third passage of SVV isolate induced typical cytopathic effects, characterized by rounding, shrinkage, and degeneration of BHK-21 cells at 24 h post-infection (Figure 1A). As shown in Figure 1B, the plaque morphology in BHK-21 cells was similar in sized and was distict. Immunofluorescence assay and Western blotting analyses were performed using homemade polyclonal anti-SVV VP1 antibody. As shown in Figure 1C, cells infected by the strains reacted to the specific polyclonal antibody against SVV VP1 protein with IFA. Meanwhile, Western blotting analyses showed an approximately 35 kilodalton (kDa) band in cells that were infected with the isolate (Figure 1D, lane 1), but not in mock cells (Figure 1D, lane 2). These results indicated that SVV LNSY01-2017 could successfully infect BHK-21 cells and proliferate, which offered a good foundation of vaccine antigen production. 

### 2.2. Inactivation of SVV LNSY01-2017 with BEI and BPL

**Figure 2:**
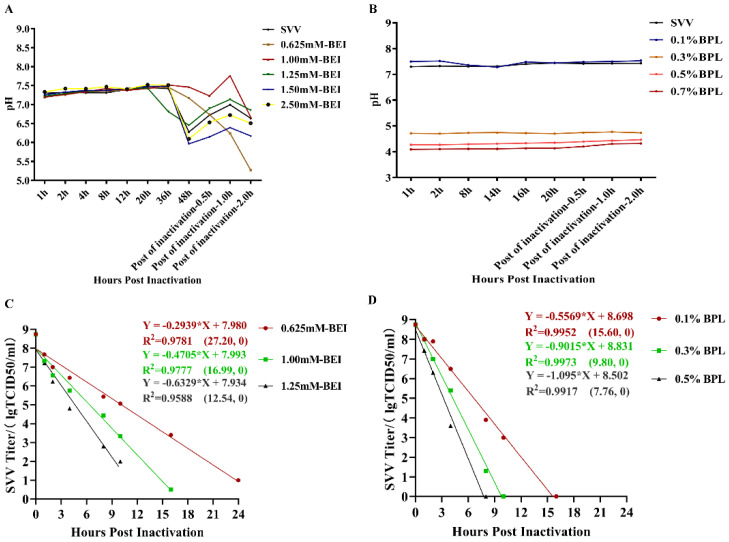
Inactivation of SVV LNSYO1-2017 with BEI and BPL. (**A**) Monitoring of pH value change with BEI concentrations. (**B**) Monitoring of pH value change with BPL concentrations. (**C**) Inactivation kinetics of different BEI concentrations. The coefficients of determinations (R^2^) of these 3 linear regressions were >0.95. (**D**) Inactivation kinetics of different BPL concentrations. The coefficients of determinations (R^2^) of these 3 linear regressions were >0.99.

*Picornaviridae* viruses are sensitive to changes in environmental pH. SVV is more inclined to “uncapsid” in an acidic environment and release nucleic acid, and its structural protein forms unstable “spent particles” [21]; the neutrality of the inactivated environment may be a key factor in the stable existence of SVV. To evaluate pH changes and the inactivation efficiency of BEI and BPL in the process of inactivating SVV, monitored pH value changes and drew inactivation curves during the process of inactivation. A series of final concentrations for BEI and BPL were determined to inactivate SVV. The final concentrations of BEI were set as 0.625 mM–2.50 mM. The inactivation time was set to 48 h and the inactivation temperature was set to 30 °C. Then, termination of inactivation was set as 2 h at 37 °C. As shown in Figure 2, at 0–20 h post inactivation with BEI, the pH values of all the different concentrations of inactivated samples were basically unchanged, ranging from 7.0 to 7.5, including the control. Twenty hours post inactivation, the pH of the inactivated samples with a BEI concentration of 1.25 mM dropped to around 6.5 and the pH of the other inactivated samples started to drop at 36 h post inactivation. The inactivated samples with 1.25, 1.50, and 2.50 mM BEI dropped to 6.0–6.5 at 48 h post inactivation. The pH of the inactivated samples with 0.625 mM BEI dropped from 36 h post inactivation until termination inactivation, and ranged from 7.3 to 5.2. Subsequently, we further monitored the BEI inactivation curve; according to the linear regression analysis results, the times required for a complete inactivation of SVV with BEI concentrations of 0.625 mM, 1.25 mM, and 2.5 mM at 30 °C ranged 24.45–30.73, 15.13–19.54, and 10.44–16.14 h. Meanwhile the pH value of different BPL concentrations showed different results, as shown in Figure 2B. Different BPL concentrations maintained a stable pH value during the entire inactivation period. Concentrations of 0.1% BPL maintained the pH value at 7.1–7.3, which was similar to SVV without BPL. However, the pH values of the other BPL concentrations (0.3%, 0.5%, and 0.7%) were maintained at 4.7–4.8, 4.1–4.2, and 4.0–4.1. The predicted times required for absolutely inactivation with BPL concentrations of 0.1%, 0.3%, and 0.5% at 4 °C ranged were 14.78–16.58, 9.34–10.31, and 6.95–8.86 h (Figure 2D). The inactivation rates of SVV were correlated with the BEI and BPL concentration, treatment time, and contact temperature. 

### 2.3. Detection Antigen (VP1) of BEI/BPL Inactivated Samples

The capsid protein is usually the main antigen component in inactivated viral vaccines, especially in *Picornaviridae* family viruses. To determine the antigens of inactivated samples, Western blotting analyses were conducted using homemade rabbit polyclonal anti-SVV VP1 antibody, as shown in Figure S1. By increasing the inactivation times of BEI, the contents of VP1 were reduced in a time-dependent fashion. While, BEI did not reduce VP1 contents in a concentration-dependent fashion, the content of VP1 in the 1.25 mM BEI concentration was the highest among 0.625 mM, 1.25 mM, and 2.50 mM concentrations (Figure S1A). Moreover, we further detected the antigens of the VP1 content in samples collected from determined hours post inactivation, for BEI concentrations stored for 7 and 14 days at 4 °C (Figure S1B,C). The VP1 contents of the 1.25 mM BEI concentration was slightly higher than that of the 0.625 mM and 2.50 mM BEI concentrations, which were stored for 7 days at 4 °C (Figure S1B). After being stored for 14 days at 4 °C, the contents of VP1 of the collected samples were slightly reduced compared with samples stored for 7 days at 4 °C. Moreover, the contents of VP1 for the 0.625 mM BEI concentrations at 6, 24, 36, 48, and 60 h were higher than those of the 1.25 mM and 2.5 mM BEI concentrations, except for the 9 h sample (Figure S1C). 

The VP1 contents of samples with different BPL concentrations were also analyzed using Western blotting (Figure S1D,E). By increasing the inactivated concentrations of BPL, VP1 content was reduced in a concentration-dependent fashion. The VP1 content was not reduced in a time-dependent fashion. At 4–10 h post inactivation, VP1 content was reduced with an increasing inactivation times, while the VP1 contents for 16 h post inactivation was slightly higher than those of 4 h, 6 h, and 10 h. Similarly, we detected VP1 contents for 0.1% BPL inactivated samples stored for 7 days and 14 days at 4 °C, the VP1 contents of the different inactivated time collections were reduced slightly compared with the SVV LNSY01-2017 control, which maintained at a better status than that of the BEI inactivated samples. According to the results above, BPL was selected as the SVV inactivator, and the inactivation concentration was set to 0.1%.

### 2.4. Immune Response of the SVV LNSYO1-2017 Inactivated Vaccines in Pigs

**Figure 3:**
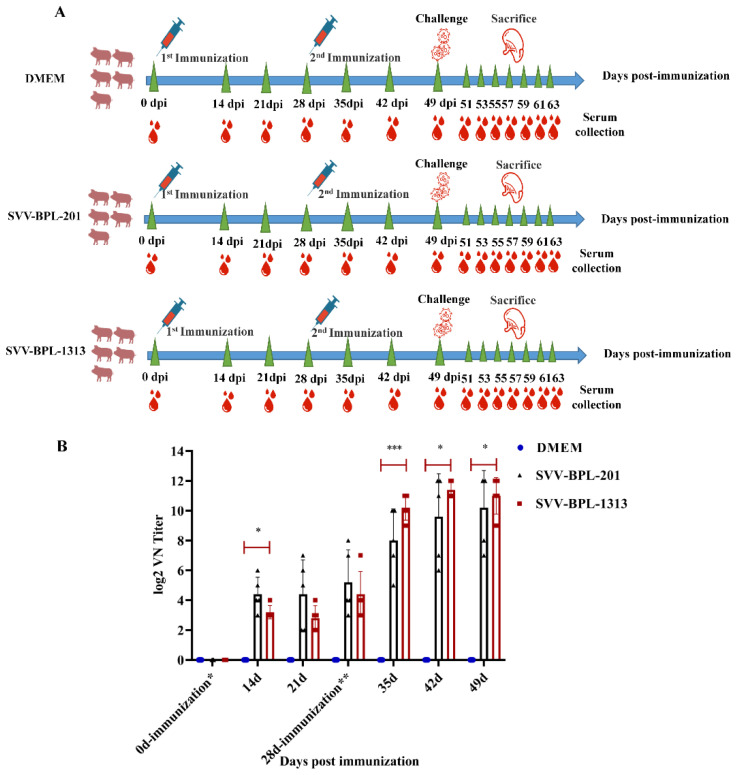
Humoral responses of pigs with SVV LNSYO1-2017 inactivated vaccines. (**A**) Immunization and challenge scheme. (**B**) Neutralizing antibody responses of pigs during the immunization period. Data represent group means ± SD. Significance was determined using ANOVA (* *p* < 0.05; *** *p* < 0.001).

**Figure 4:**
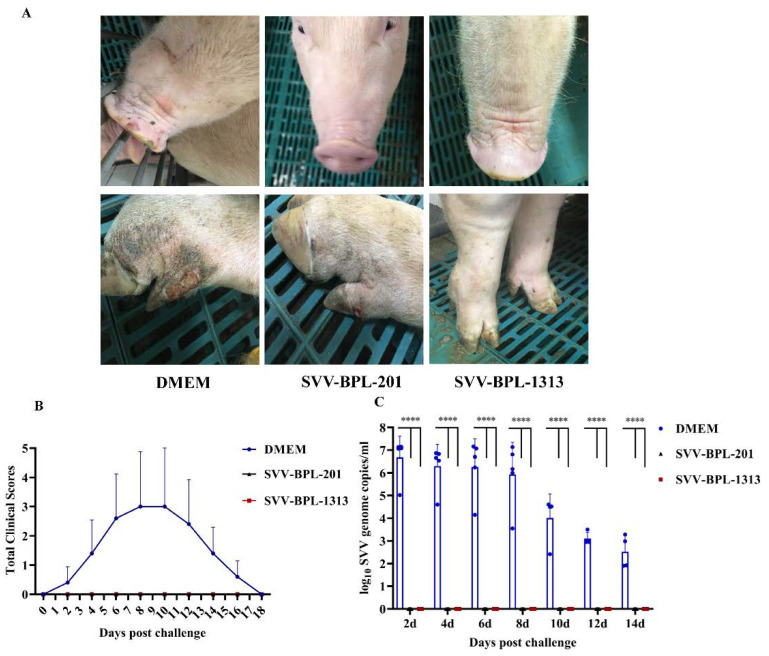
Vesicular lesion, clinical scores, and viremia of pigs, post infection challenge. (**A**) Vesicular lesion observed on pigs in the immune group and control group, challenged with SVV LNSYO1-2017. (**B**) Clinical scores of the pigs challenged with SVV LNSYO1-2017. Data represent the mean ± SD. (**C**) Detection of the viremia levels in the serum of pigs challenged with SVV LNSYO1-2017. Data represent the mean ± SD. Significance was determined using ANOVA (**** *p* < 0.000001).

Fifteen six-week-old finishing pigs were randomly divided into three groups, which were negative with PCR detection of in neutralizing antibody tests before immunization. Two groups (immunized with SVV-BPL-201 or SVV-BPL-1313) were the immune groups the third group (immunized with DMEM) was the control group. The immunization period lasted for 49 days and the first immunization was set at day 0 and the second immunization was at 28 days. Twelve hours after being immunized, the temperatures of the pigs in the SVV-BPL-201 and SVV-BPL-1313 groups reached 39.5 °C to 39.7 °C (a temperature from 38.5 °C to 40.0 °C is considered normal for pigs). Then, the temperatures of pigs in the SVV-BPL-201 and SVV-BPL-1313 groups showed a downstream trend; overall, the temperature in all groups undulated in status in the normal range (data not shown). Neutralizing antibody (NA) titers were detected in the serum samples that were collected from the pigs. The results shown in Figure 3, post first immunization, indicate that the NA titers of the immune groups increased slightly and were mainly concentrated at 1:4–1:64. The NA titers of individual pigs reached 1:128–1:256. The NA level of SVV-BPL-201 was higher than that of BPL-1313 before the second immunization. The NA level of the immune groups increased sharply and substantially post second immunization. In particular, the NA titers of the SVV-BPL-1313 group remained at 1:512–1:4096 until 21 days post second immunization (Figure 4B). After the second immunization, the NA level for SVV-BPL-1313 was higher than that of SVV-BPL-201. No NA titers were detected in the DMEM group.

### 2.5. Vesicular Lesion and Clinical Scores Observed in Pigs Post-Challenge Infection

Based on the results above, we further evaluated the protective efficacy of the SVV LNSY01-2017-inactivated vaccine. Pigs were challenged with 3 mL (10^9.5^ TCID_50_/_mL_) of SVV LNSY01-2017 virus via nasal inhalation at 49 days post immunization (dpi). After being challenged, clinical lesions and scores were monitored and recorded. The clinical scores were used to evaluate vesicular lesions following a previously established method [19]. Clinical signs were scored as follows: no symptoms appeared, 0 point; each foot bearing lesions, 1 point; vesicular lesions in or around the mouth, 1 point. Therefore, the maximum score per animal was 5. As shown in Figure 4A,B, 4 of 5 pigs in the DMEM group developed clinical signs at 2–4 days post challenge (dpc); vesicular lesions developed on the lips and hooves and then broken blisters were found, which limited activity and reduced appetite. Peak clinical scores were mainly observed at 8–10 dpc, and clinical symptoms disappeared at 16–18 dpc. Both pigs from the immune groups did not present clinical symptoms of SVV. Clinical scores for the SVV-BPL-201 and SVV-BPL-1313 groups remained at 0 throughout the challenge phase of the experiment. RNAemia/SVV viremia was assessed 2–14 dpc (Figure 4C). In the DMEM group, SVV viremia maintained a high level, 2–8 dpc, with the highest viremia levels being detected 2 dpc (10^6.687^ copies/mL), remaining elevated until 8 dpc (10^5.929^ copies/mL), and then reducing immediately. The SVV viremia in the DMEM group was still detected 14 dpc (10^3.023^ copies/mL). In contrast, the SVV viremia of the SVV-BPL-201 and SVV-BPL-1313 groups was not found during the entire challenge period. 

### 2.6. Histopathological Analysis of Post-Challenge Infection

**Figure 5:**
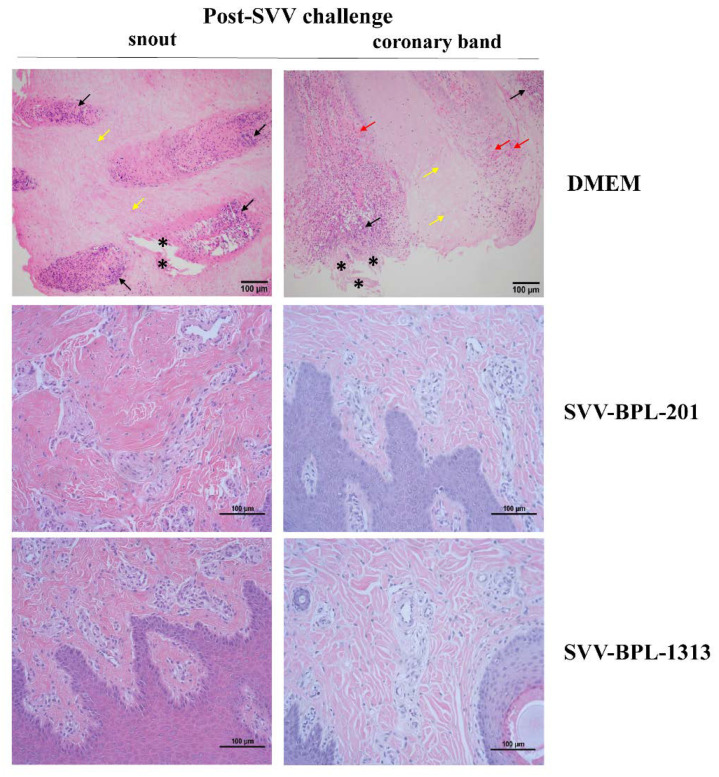
Histopathological analysis of pigs, post-challenge infection. Histopathological examination of the tissue of pigs challenged with the SVV LNSYO1-2017 strain. Original magnification, 200×.

We described the pathogenic differences amongst pigs in the immune groups and the DMEM group during the virus challenge period. Three pigs were selected from the three groups and were killed humanely at 8 dpc. Histology was used to describe the pathologic damage to multiple organs, including the spleen, mesenteric lymph node, inguinal lymph node, snout, and coronary band. In the DMEM group pig, the overall structure of the spleen was abnormal. Obvious abnormal hyperplasia of fibrous connective tissue was observed. Congestion occurred in the cortex and there was a sharp reduction in lymphocytes accompanied by an obvious increase in red pulp (data not shown). Notably, significant epithelial cell necrosis and necrotic foci caused by SVV infection were observed in the snout and coronary band of pigs in the DMEM group (Figure 5). Most of the necrotic area showed the phenomenon of cell nucleus shrinkage, dissolution, and disappearance, accompanied by cellular vacuolar degeneration (yellow arrow). Numerous neutrophil infiltrations occurred and a number of fragments from inflammatory cells were observed in the tissues (black arrow). Furthermore, in part of the necrotic area, epithelial cells were obviously shed and the shapes were severely deformed (black star). Moreover, obvious bleeding was observed in the coronary band (red arrow). No histopathological lesions were observed in the snout or coronary band for the SVV-BPL-201 and SVV-BPL-1313 groups. 

## 3. Discussion

Continuous circulation, spread, and evolution of SVV has led to an urgent demand for effective vaccines. Numerous vaccines have been found to be safe and effective for the prevention of diseases caused by viruses and bacteria, such as the influenza virus, poliovirus, SARS-CoV-2, and whole-cell Bordetella pertussis [22,23,24,25]. As is well known, FMDV is effectively controlled around the world through the use of inactivated vaccines [26]. SVV is similar to FMDV in terms of biological characteristics, and some strategies for the development of inactivated vaccines can be optimized from already approved strategies. BEI and BPL are two inactivating agents with excellent inactivation effects and are widely used. BEI is widely used for the FMDV-inactivated vaccine [27,28]. Beta-propiolactone (BPL) is a reagent that is commonly used for virus inactivation in vaccine preparations [29,30,31]. BPL can inactivate a virus at 4 °C; moreover, when inactivation needs to be terminated, BPL can be hydrolyzed at 37 °C without residuals [32]. We evaluated the inactivated condition and efficiency of BEI and BPL by monitoring pH variation and the inactivation curve during the inactivation process. The two inactivating agents showed a good linear relationship in inactivating SVV. Monitoring the pH variation during the inactivation process showed that the pH value of the SVV inactivated samples, with different BEI concentrations, could be kept stable until SVV was completely inactivated. The pH value of the SVV inactivated samples with different BPL concentrations remained stable, the pH of the 0.1% BPL-inactivated samples was close to that of the untreated SVV group, and the pH values of the other concentration groups were lower than that of the untreated SVV group. An earlier study found that the structure of virus particles is sensitive to pH changes. Under low pH conditions, virus particles are depolymerized into pentamers to facilitate genome uncoating [21]. The occurrence of pentamers may affect the stability and functional activity of a viral capsid protein. It is necessary to maintain the pH for SVV inactivation as neutral. The detection of VP1 content in the collections with BEI for different inactivation times showed that the VP1 contents of the 1.25 mM BEI concentration were closest to those of the untreated SVV group. The VP1 contents of the inactivated samples of different BEI concentrations stored for 6–7 days and 13–14 days at 4 °C were further detected. VP1 degradation was obvious in the 2.5 mM and 0.625 mM BEI-inactivated samples. The contents of VP1 for the 1.25 mM BEI concentration was close to that of the untreated SVV group. Meanwhile, with different BPL inactivation concentrations, VP1 contents were different. VP1 degradation was severe for 0.3–0.5% BPL, while a VP1 content under 0.1% BPL was similar to that of untreated SVV and the VP1 content of 0.1% BPL-inactivated samples stored for 7 and 14 days was still similar to the VP1 contents of untreated SVV. It is speculated that the acidic environment, caused by 0.3–0.5% BPL, affects the stability of the SVV protein. 

The evaluation of neutralizing antibodies induced by SVV-inactivated vaccines showed that neutralizing antibodies can be detected in the immunized group at 7 dpi, with a higher level of SVV-BPL-201 detected before the second immunization, followed by SVV-BPL-1313. The overall level of neutralizing antibodies is not high. Post second immunization (28 dpi), the neutralizing antibodies in the immune group increased rapidly and the level of neutralizing antibodies in the SVV-BPL-1313 group was higher (a mean titer of 1:2048 compared with SVV-BPL-201 that had a mean titer of 1:1024). Moreover, the neutralizing antibody of the SVV-BPL-1313 group remained high after the second immunization. It is reasonable to think that the SVV-BPL-1313 group induced a better humoral immune response in pigs. In this study, the immune group could not induce a rapid humoral immune response until the second immunization, which should be investigated further.

At 49 dpi, all pigs were challenged with the pathogenic SVV LNSY01-2017 strain to evaluated the protection efficacy. The SVV viremia of pigs in the DMEM group was detected post challenge, with the highest viremia levels detected at 2 dpc, remaining elevated until 8 dpc. Then, here was an immediate reduction in SVV viremia. SVV viremia was detected until 14 dpc. Related to this, pigs in the DMEM group showed clinical signs of SVV at 14 dpc and did not fully recover until 18 dpc (Figure 4B). In contrast, no SVV viremia was detected in the serum of the SVV-BPL-201 and SVV-BPL-1313 groups during the study. Our findings are consistent with previous reports [19,33]; pigs with a good humoral immune response can prevent the development of SVV viremia.

The pigs in the DMEM group showed ulceration in hoof tissues, blisters in snout tissues, and other clinical symptoms from 2–4 dpc. These symptoms were most severe 6–8 dpc and 80% (4/5) of pigs in the DMEM group showed typical symptoms. Further pathological section results showed that the hoof and snout tissues from the DMEM group showed pathological phenomena, such as necrosis, shedding, bleeding, and ballooning degeneration of epithelial cells, caused by acute SVV infection. Pathological phenomena, such as a violent reduction in leukomonocytes, abnormal hyperplasia of fibrous connective tissue, and congestion, were also observed in the spleen. These results suggested that the inactivated vaccine provided good immune protection for the immunized groups of pigs.

In recent years, some studies have tested the efficacy of SVV-inactivated vaccines. Yang [19] first reported that SVV-inactivated vaccines can defend against challenge by SVV in finishing pigs. Further, Yang evaluated the antibody response of sows after vaccination with an SVV-inactivated vaccine as well as the effects of maternal antibody transfer on antibody dynamics in offspring without a challenge test for the piglets to determine if their titers were protective [34]. Yang used a purified SVV virus as an immunogen in his study. Purifying the virus is beneficial to enrich antigens, but the associated high cost may be unavoidable and is inconvenient for large-scale production. Meanwhile, the processes of concentration and purification take a long time and require an extensive operations, which may cause certain losses in terms of the virus. Yang used a BCA method to measure the protein content in an inactivated solution and BCA measured the total protein content, not the specific antigens of SVV in inactivated samples. The aim of quantifying antigen content via total protein concentration is to obtain high-purity virus particles, which may also increase the difficulty of antigen preparation. In another study [35], Fan successfully constructed a His-tagged SVA mutant rSVA-His that stably expresses 6× His-tag on the surface of an SVA particle, and the rSVA-His can be used for rapid purification of SVA antigens for inactivated vaccines. This study provides another feasible strategy for purifying SVV. Li [36] developed an SVV-inactivated vaccine using BPL as the inactivator that can protect finishing pigs against challenge by the homologous virus. This is the first report of the application of BPL to inactivate SVV. The abovementioned studies offer promising inactivated vaccine candidates; however, these studies could not fully demonstrate the specific process and changes in antigen content during inactivation. SVV is easily “destabilized” in an acidic environment [21]. Different treatments with inactivators will inevitably affect the stability of SVV structural proteins. Considering the characteristics of the two inactivators and the acid–labile characteristics of the SVV virus, it is necessary to explore the effects of different inactivators on SVV. Herein, we comprehensively compared the inactivation effects of BEI and BPL on SVV by monitoring pH change, the inactivation curve, and the content change of VP1 in the inactivated samples during and post inactivation process. The results showed that the inactivation effect of BPL was better than that of BEI. Our findings provide intuitive evidence that different inactivating agents have different effects on SVV inactivation. However, determination of the optimal concentrations of BPL for inactivation, improved vaccine stability and shelf life, optimization of the pathogenesis model, and more comprehensive analysis of immune indicators, including the evaluation of humoral immunity and cellular immunity of pigs after vaccine immunization, are needed for the development of SVA-inactivated vaccine.

## 4. Conclusions

Our study investigated an SVV-inactivated vaccine based on screening of inactivators (BEI and BPL) and adjuvants of ISA 201 and IMS 1313. The data suggested that the inactivation effect of BPL was better than that of BEI, and the SVV LNSY01-2017 (BPL-1313)- inactivated vaccine had good immunoreactivity and protection efficacy. Optimization of vaccine production will be necessary to induce a quick and high level of immune response after the first immunization.

## Data Availability

The data presented in this study are available upon request from the corresponding author.

## Supplementary Materials

The following supporting information can be downloaded at: https://www.mdpi.com/article/10.3390/vaccines10040631/s1, Figure S1: The content of antigen (VP1) detection of BEI- and BPL-inactivated samples. (A) Detection of VP1 content of different BEI concentrations at different hours post inactivation. (B) Detection of VP1 content of inactivated samples with different BEI concentrations stored for 6–8 d at 4 °C. (C) Detection of VP1 content of inactivated samples with different BEI concentrations stored for 13–15 days at 4 °C. (D) Detection of VP1 content of inactivated samples with different BPL concentrations at different hours post inactivation. (E) Detection of VP1 content of inactivated samples with different BEI concentrations stored for 7 days and 14 days at 4 °C. Table S1: log10 SVV genome copies/ml total RNA read in Figure 4C.
