# Supplementary material for: Evaluation of Immunoreactivity and Protection Efficacy of Seneca Valley Virus Inactivated Vaccine in Finishing Pigs Based on Screening of Inactivated Agents and Adjuvants
Source: Vaccines (Basel). 2022 Apr 18;10(4):631. doi: 10.3390/vaccines10040631 (PMC9032702; doi:10.3390/vaccines10040631)
Supplement: Supplementary file 1 [file vaccines-10-00631-s001.zip › vaccines-1649029-supplementary.pdf]

Supplementary Information for

Evaluation of immunoreactivity and protection efficacy of Seneca Valley virus inactivated vaccine in finishing pigs based on screening of inactivated agents and adjuvants

Wenqiang Liu[1,2], Xiangmin Li [1,2,3,4], Huawei Zhang[1,2], Genxi Hao[1,2], Xianfei Shang[1,2], Huilan Wang[1,2], Huanchun Chen [1,2,3,4] and Ping Qian [1,2,3,4*]

Supplementary information includes 1 Figure.

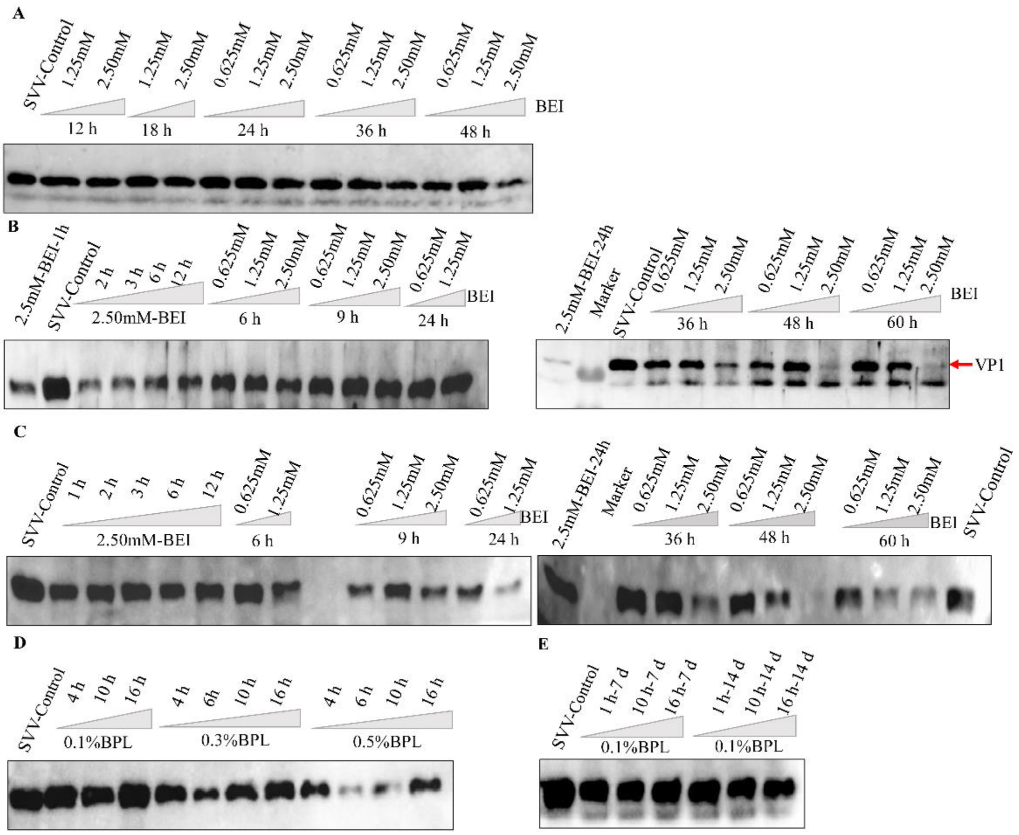

Figure Supplement 1. The content of antigen (VP1) detection of BEI and BPL inactivated samples. (A) Detection of VP1 content of different BEI concentrations in different hours post inactivation. (B) Detection of antigen VP1 of inactivated samples with different BEI concentrations stored at 4℃ for 6-8 d. (C) Detection of VP1 content of inactivated samples with different BEI concentrations stored at 4℃ for 13-15 d. (D) Detection of VP1 content of

inactivated samples with different BPL concentrations in different hours post inactivation. (E) Detection of VP1 content of inactivated samples with different BEI concentrations stored at 4℃ for 7 d,14

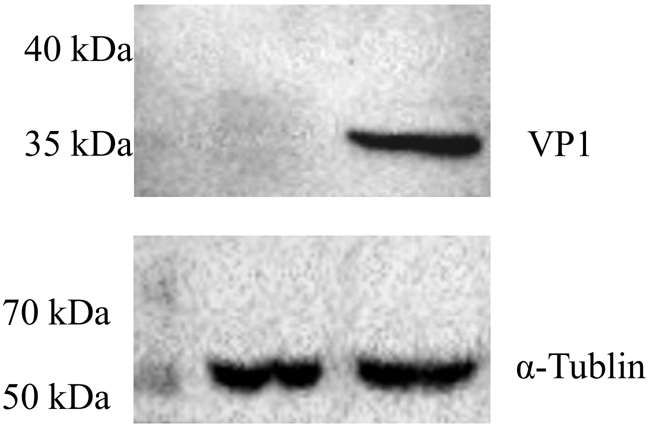

	lane 1	lane 2
Relative raw Intergrated density	1	1.000829
Raw Intergrated density	1988267	1989915

Figure S1. The blot (uncropped blots) showing all the bands with molecular weight markers of figure 1D is.

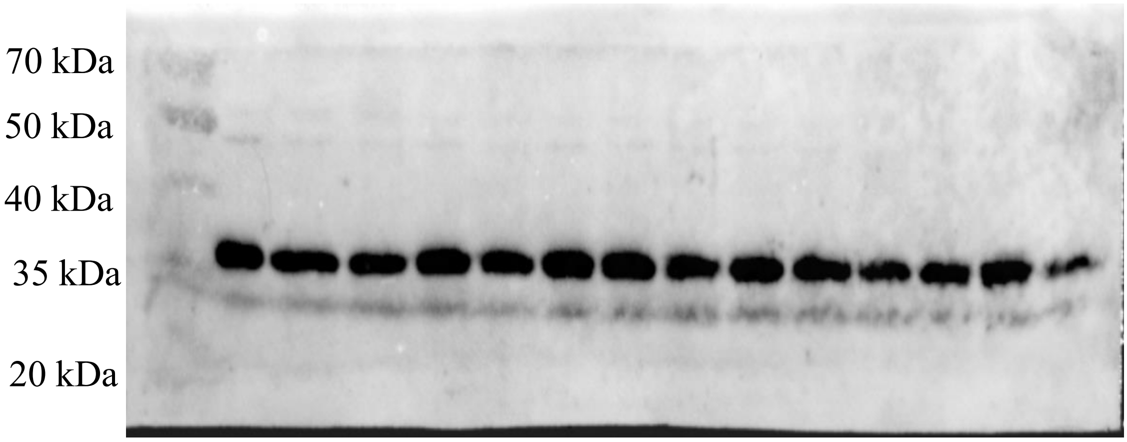

	lane 1	lane 2	lane 3	lane 4	lane 5	lane 6	lane 7
Relative raw Integrated density	1	1.242275	1.048989	1.114306	1.07117	1.1445	1.159405
Raw Integrated density	1490453	1851552	1563469	1660821	1596528	1705823	1728039

	lane 8	lane 9	lane 10	lane 11	lane 12	lane 13	lane 14
Relative raw Integrated density	0.990308	1.122923	1.1244	0.848949	0.961165	1.114616	0.61567
Raw Integrated density	1476008	1673664	1675865	1265319	1432571	1661283	917627

Figure S2. The blot (uncropped blots) showing all the bands with all molecular weight markers of figure supplementary 1A is.

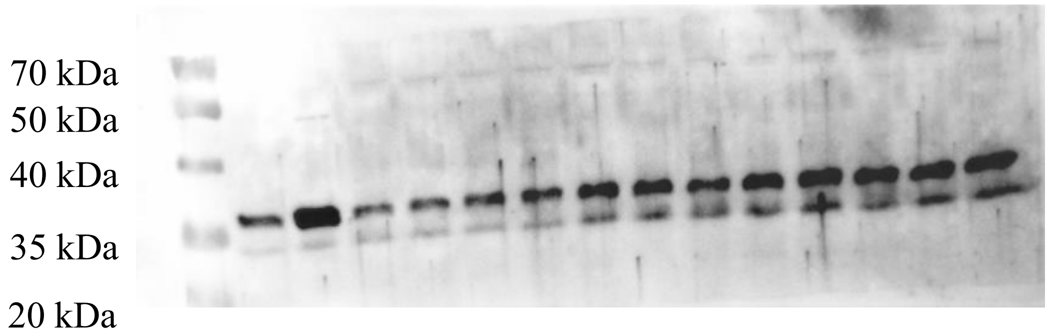

	lane 1	lane 2	lane 3	lane 4	lane 5	lane 6	lane 7
Relative raw Integrated density	0.469138	1	0.41144	0.448398	0.539594	0.654253	0.751427
Raw Integrated density	64422	137320	56499	61574	74097	89842	103186

	lane 8	lane 9	lane 10	lane 11	lane 12	lane 13	lane 14
Relative raw Integrated density	0.745012	0.680629	0.724396	0.831998	0.85327	0.791924	0.932311
Raw Integrated density	102305	93464	99474	114250	117171	108747	128025

Figure S3. The blot (uncropped blots) showing all the bands with all molecular weight markers of figure supplementary 1B（left）is.

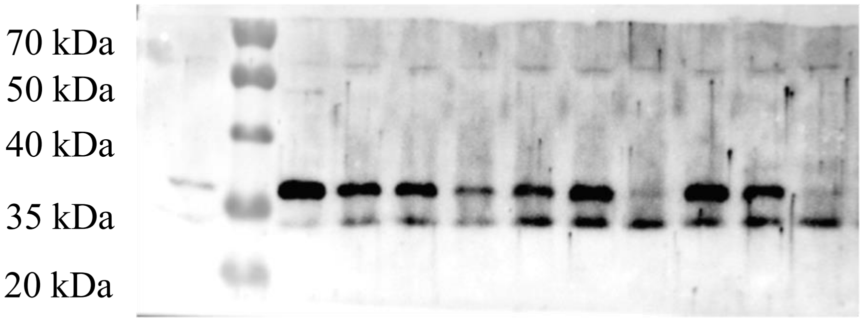

	lane 1	lane 2	lane 3	lane 4	lane 5	lane 6	lane 7
Relative raw Integrated density	0.055185	1	0.764696	0.732081	0.528303	0.751953	0.922281
Raw Integrated density	7609	137881	105437	100940	72843	103680	127165

	lane 8	lane 9	lane 10	lane 11
Relative raw Integrated density	0.420725	0.983486	0.729687	0.358367

Raw Integrated density	58010	135604	100610	49412

Figure S4. The blot (uncropped blots) showing all the bands with all molecular weight markers of figure supplementary 1B（right）is.

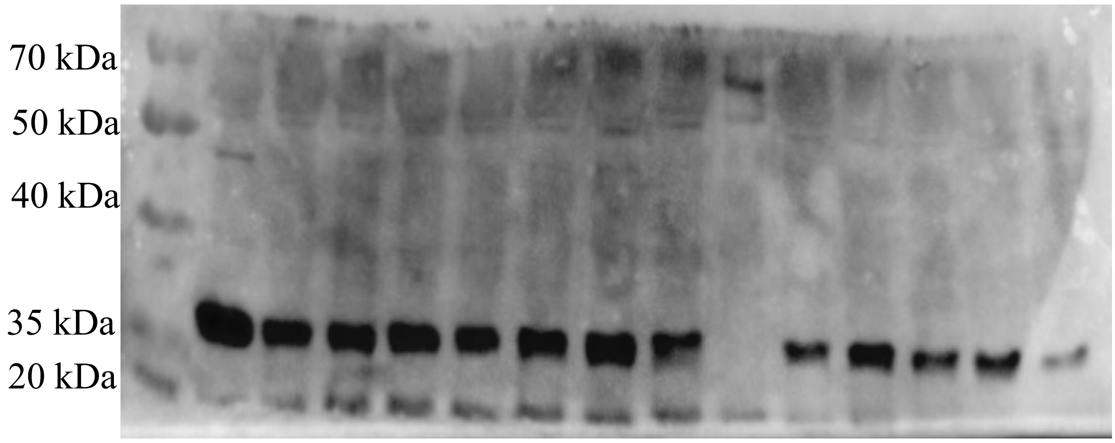

	lane 1	lane 2	lane 3	lane 4	lane 5	lane 6	lane 7
Relative raw Intergrated density	1	0.50236	0.425451	0.490043	0.373358	0.525437	0.641334
Raw Intergrated density	1665259	836559	708486	816048	621738	874989	1067987

	lane 8	lane 9	lane 10	lane 11	lane 12	lane13
Relative raw Intergrated density	0.418821	0.273965	0.474393	0.27175	0.379766	0.298984
Raw Intergrated density	697446	456222	789988	452534	632409	497886

Figure S5. The blot (uncropped blots) showing all the bands with all molecular weight markers of figure supplementary 1C (left) is.

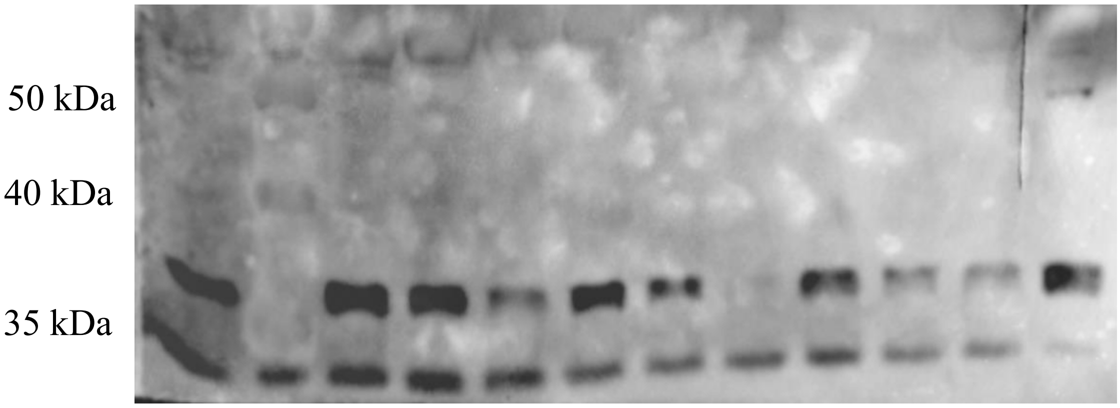

	lane 1	lane 2	lane 3	lane 4	lane 5	lane 6	lane 7
Relative raw Integrated density	0.469124	0.903839	0.941265	0.442334	0.767099	0.527696	0.661988
Raw Integrated density	466993	899733	936989	440324	763614	525299	658981

	lane 8	lane 9	lane 10
Relative raw Integrated density	0.25625	0.257835	1
Raw Integrated density	255086	256664	995457

Figure S6. The blot (uncropped blots) showing all the bands with all molecular weight markers of figure supplementary 1C (right) is.

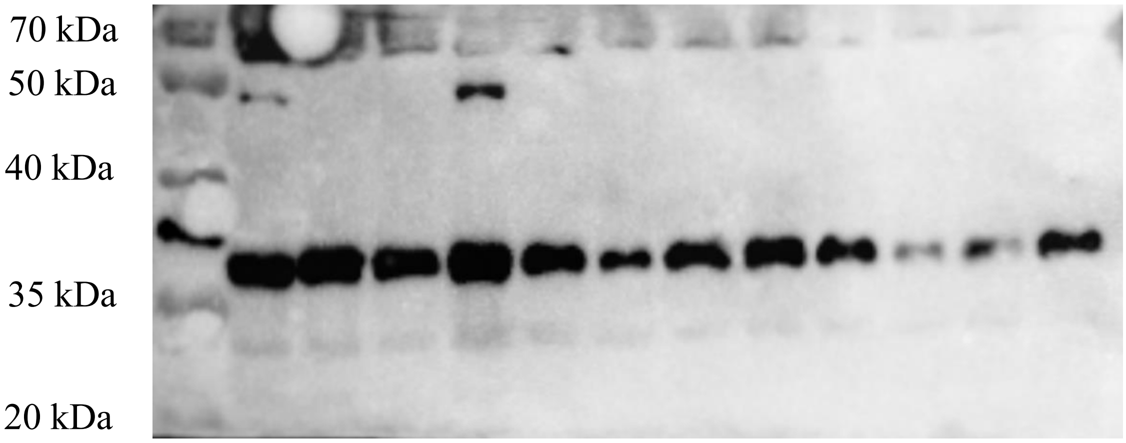

	lane 1	lane 2	lane 3	lane 4	lane 5	lane 6	lane 7
Relative raw Integrated density	1	1.009553	0.846337	1.02203	0.77181	0.405494	0.646221
Raw Integrated density	4401438	4443483	3725102	4498401	3397072	1784757	2844302

	lane 8	lane 9	lane 10	lane 11	lane 12
Relative raw Integrated density	0.656142	0.460216	0.073925	0.206977	0.458465
Raw Integrated density	2887970	2025612	325375	910996	2017906

Figure S7. The blot (uncropped blots) showing all the bands with all molecular weight markers of figure supplementary 1D is.

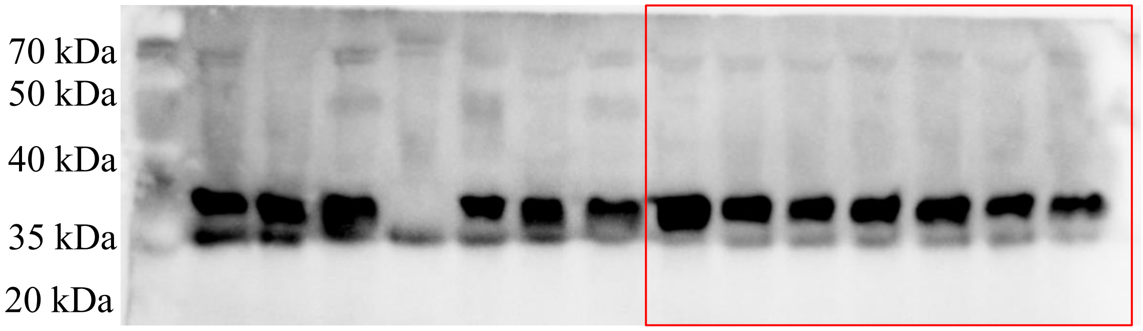

	lane 1	lane 2	lane 3	lane 4	lane 5	lane 6	lane 7
Relative raw Integrated density	1	0.731011	0.602422	0.695434	0.754378	0.57405	0.514068
Raw Integrated density	3654865	2671746	2201770	2541718	2757151	2098074	1878848

Figure S8. The blot (uncropped blots) showing all the bands with all molecular weight

markers of figure supplementary 1E is.

Table S1. log10 SVV genome copies/ml total RNA read in Figure 4C is

Days post challenge	log10 SVV genome copies/ml Group		
	Control	BPL-201	BPL-1313
2d	5.023298;7.077215; 7.062168;7.129689; 7.14379	0; 0; 0; 0; 0	0; 0; 0; 0; 0
4d	4.600745;6.540895; 6.658265;6.86633; 6.83127	0; 0; 0; 0; 0	0; 0; 0; 0; 0
6d	4.149201;6.241659; 7.155992;7.068536 6.704075	0; 0; 0; 0; 0	0; 0; 0; 0; 0
8d	3.544638; 6.807146 7.130786; 6.167643 5.994349	0; 0; 0; 0; 0	0; 0; 0; 0; 0
10d	2.412735;4.604300 4.502354 4.518465	0; 0; 0; 0; 0	0; 0; 0; 0; 0
12d	2.94780; 3.4981600 2.955026;3.021415	0; 0; 0; 0; 0	0; 0; 0; 0; 0
14d	1.903245; 3.2783 1.923475; 2.976532	0; 0; 0; 0; 0	0; 0; 0; 0; 0
